# Fabrication of Microgel-Modified Hydrogel Flexible Strain Sensors Using Electrohydrodynamic Direct Printing Method

**DOI:** 10.3390/s24103038

**Published:** 2024-05-10

**Authors:** Junyan Feng, Peng Cao, Tao Yang, Hezheng Ao, Bo Xing

**Affiliations:** 1 College of Mechanical and Electronic Engineering, Jiaxing Nanhu University, Jiaxing 314001, China; 2 College of Mechanical Engineering, Zhejiang University of Technology, Hangzhou 310014, China; 2112102418@zjut.edu.cn (T.Y.); 2112102451@zjut.edu.cn (H.A.); 3 College of Information Science and Engineering, Jiaxing University, Jiaxing 314000, China; xingbo@zjxu.edu.cn

**Keywords:** hydrogel, flexible strain sensors, electrohydrodynamic, electrochemical properties

## Abstract

Hydrogel flexible strain sensors, renowned for their high stretchability, flexibility, and wearable comfort, have been employed in various applications in the field of human motion monitoring. However, the predominant method for fabricating hydrogels is the template method, which is particularly inefficient and costly for hydrogels with complex structural requirements, thereby limiting the development of flexible hydrogel electronic devices. Herein, we propose a novel method that involves using microgels to modify a hydrogel solution, printing the hydrogel ink using an electrohydrodynamic printing device, and subsequently forming the hydrogel under UV illumination. The resulting hydrogel exhibited a high tensile ratio (639.73%), high tensile strength (0.4243 MPa), and an ionic conductivity of 0.2256 S/m, along with excellent electrochemical properties. Moreover, its high linearity and sensitivity enabled the monitoring of a wide range of subtle changes in human movement. This novel approach offers a promising pathway for the development of high-performance, complexly structured hydrogel flexible sensors.

## 1. Introduction

With the rapid development of flexible electronics in recent years, the interest in and demand for flexible wearable devices have been steadily increasing [1,2,3,4,5,6,7]. Consequently, flexible, stretchable, and human-friendly strain sensors have attracted considerable attention in the fields of human motion detection and health monitoring. Currently, strain sensors are commonly prepared by coating or dispersing metallic materials, carbon-based materials, or conductive polymer fillers into elastomers such as rubber or polyurethane [8,9,10]. Although these nanofillers enhance electrical conductivity, the phase separation between nanoparticles and the elastomer material during the operation of the strain sensors may cause the nanofillers to detach during use, ultimately resulting in sensor failure [11,12].

Hydrogels are flexible materials characterized by a unique three-dimensional network structure formed through the physical or chemical cross-linking of polymers, offering excellent tensile properties and elasticity [13]. Owing to their highly customizable structure and functionality, hydrogels have been employed in various applications in biomedicine, e-skin, soft robotics, and wearable sensors [14,15,16,17,18]. However, current hydrogel preparation methods often involve complex and time-consuming mold transfer processes, requiring specialized molds tailored to hydrogels and suitable mold-casting methods, which severely limit the diversity of hydrogel structures [19,20]. Therefore, developing stretchable and conductive hydrogels with 3D printability is crucial.

Considerable research has been conducted on the 3D printing of hydrogels [21,22]. However, to achieve specific properties, the viscosity and rheological properties of hydrogel solutions are often unsuitable for printing. Because high-viscosity inks exhibit shear-thinning behavior, most current methods involve increasing the viscosity of hydrogel inks to make them suitable for printing on various shapes [23,24,25]. However, high-viscosity solutions can lead to slumping during the printing process, significantly reducing the printing accuracy. Microgels are internally cross-linked macromolecules that have been employed as rheology modifiers for various 3D printing inks and are effective alternatives to high-viscosity hydrogel inks [26,27,28,29,30]. Clogged microgels exhibit excellent thixotropic properties, with the microparticles held in place by physical interactions, resulting in solid-like behavior. When subjected to sufficient extrusion stress, these physical interactions break, causing the microgels to flow like a liquid. Subsequently, they revert back to a solid-like state when the stress dissipates, which significantly improves the molding ability of the hydrogel solution after printing [31,32,33]. Furthermore, microgels can serve as the primary rigid network in the hydrogel, while the monomers in the solution form the secondary network after polymerization. Consequently, this dual-network structure can effectively enhance the mechanical properties of the hydrogel.

In this study, a green and low-cost carbomer 940 powder was incorporated into a hydrogel solution to impart solid-like behavior, and the solution was patterned and printed using an electrohydrodynamic (EHD) printing device. Acrylic acid (AA) and acrylamide (AM) were polymerized under UV illumination to form Car-P(AA-co-AM) bi-network hydrogels, and the optimal ratio of monomers to microgels was determined. The electrical properties of the hydrogels were systematically examined, and the hydrogel sensors exhibited high sensitivity and linearity, capable of monitoring various human behaviors. In addition, a flexible mesh hydrogel strain sensor was fabricated, which enhanced breathability and comfort during human movement.

## 2. Materials and Methods

### 2.1. Materials

The experimental materials included ethylene glycol (EG, AR) (Guangdong Guanghua Stock Science and Technology Co., Ltd., Shantou, China), polydimethylsiloxane (PDMS), carbomer 940 powder (Carbomer 940, AR), AA (>99%), AM (AR), N,N-methylenebisacrylamide (MBA, AR), 2-hydroxy-2-methylpropiophenone (1173, 97%), and sodium chloride (NaCl, AR, 99.5%) (Shanghai McLean Biochemical Technology Co., Ltd., Shanghai, China). The carbomer was dried at 60 °C for 24 h to remove moisture.

### 2.2. Preparation of Hydrogel Precursor Solutions

Water and EG were mixed as a binary solvent at a ratio of 3:1. Different mass ratios of AA, AM (20 wt.% of AA), and the binary solvent were mixed and stirred to form a homogeneous solution. Subsequently, a certain amount of the cross-linking agent MBA (0.2 mol% of AA), photoinitiator 1173 (1 wt.% of AA), and NaCl (with a content of 0.5 mol/L in the mixed solution) were added. The mixed solution was then vigorously stirred using a magnetic stirrer for 1 h to obtain a homogeneous solution. Carbomer powders with different mass ratios (3 wt.%, 5 wt.%, and 7 wt.%) were added to the P(AA-co-AM) hydrogel precursor solution. Carbomer was slowly added into the hydrogel solution 5 times, and the solution was placed in a magnetic stirrer with a rotational speed of 800 r/min for 10 min to make the carbomer powder dispersed homogeneously, followed by stirring at a rotational speed of 300 r/min for 4 h until the carbomer powder was completely dissolved to form a homogeneous, transparent and stable solution.

### 2.3. Preparation of Hydrogel

(1) Preparation of P(AA-co-AM) hydrogels

Because the P(AA-co-AM) hydrogel precursor solution did not have the required rheological properties for 3D printing, the hydrogel was prepared using an in situ preparation method. Polylactic acid (PLA), a non-polluting green polymer material, was used as the printing material, and molds of the desired shapes were printed using an FDM 3D printer. DOWSIL SYLGARD 184a and DOWSIL SYLGARD 184b were homogeneously mixed at a mass ratio of 10:1 to form a solution, which was subsequently slowly poured into a 3D printing mold, heated, and cured by removing air bubbles through a vacuum to form a flexible PDMS mold. Subsequently, the P(AA-co-AM) hydrogel precursor solution was transferred to the PDMS mold using a syringe, and the hydrogel was formed by placing it under a UV curing lamp for 10 min.

(2) Preparation of Car-P(AA-co-AM) hydrogels

**Figure 1:**
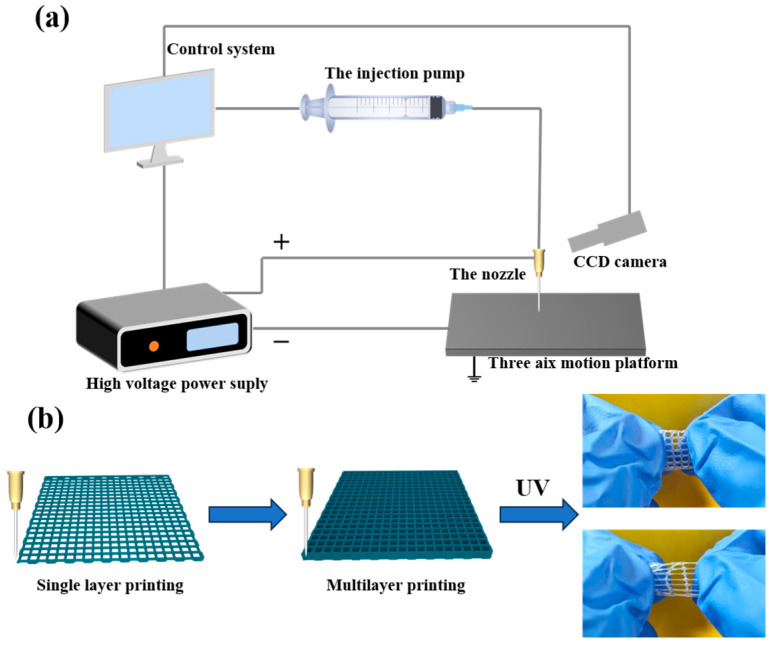
(**a**) Schematic of EHD printing equipment. (**b**) Printing and preparation of Car-P(AA-co-AM) hydrogels.

The addition of a carbomer gives the hydrogel precursor solution good thixotropic properties, meaning that it can be printed in various shapes using an EHD printing device. The main components of the EHD printing device, as shown in Figure 1a, include a three-axis motion platform, control system, injection system, high-voltage power supply, and visual monitoring system. The hydrogel precursor solution was uniformly drawn into filaments under the combined action of squeezing pressure, gravity, and an electric field force to print a thin film or grid-like hydrogel. During the printing process, a stainless steel nozzle with an inner diameter of 0.2 mm and an outer diameter of 0.31 mm served as the printing nozzle. The syringe flow rate was set at 20 μL/min, the printing height at 0.2 mm, and the printing speed at 2 mm/s. A voltage of 3 KV was applied between the substrate and the nozzle. As shown in Figure 1b, patterns of different shapes and thicknesses were printed along the print path after being drawn using the control system. The printed patterns were placed under a UV curing lamp for 10 min to promote monomer polymerization and form a hydrogel.

### 2.4. Characterization and Properties

The microscopic morphology of the freeze-dried hydrogel samples was observed using a focused ion and electron beam system (Helios 5 CX, Thermo Fisher Scientific, Waltham, MA, USA) after freeze-drying the hydrogel samples for 24 h in a vacuum freeze-dryer (LGJ-10, Beijing Songwon Huaxing Science and Technology Development Co., Ltd., Beijing, China). Changes in the correlation peaks in the infrared spectra of the hydrogels were analyzed using an in situ infrared spectrometer (Thermo Fisher IS50, Nicolet, USA).

The tensile properties of the hydrogels were assessed using a microcomputer-controlled electronic universal testing machine (DWD-0.5C, Shanghai Hualong Testing Instrument Co., Ltd., Shanghai, China) at a tensile speed of 30 mm/min, with the tensile specimens measuring 20 mm × 4 mm × 1.5 mm.

Electrochemical impedance spectroscopy (EIS) was performed on the hydrogel using an electrochemical workstation (RST 5000, Suzhou Restek Electronics Co., Ltd., Suzhou, China). The gel film was cut into a rectangular shape (15 mm × 10 mm × 1.5 mm) and sandwiched between two platinum sheets for measurement over a test frequency range of 100,000 Hz to 1 Hz and an AC amplitude of 0.007 V. The conductivity was calculated using the following equation:

(1) Σ=L/(R×S)

where *L* (cm) is the thickness of the DES gel film, *S* (cm^2^) is the contact area between the DES gel film and the platinum electrode, and *R* (Ω) is the resistance in the Nyquist diagram.

The resistance changes in the sensors at different strains were measured using a flexible electronic analysis system (AES-4 SD; Zhongju Hi-Tech, Beijing, China) at a tensile speed of 1 mm/s. The resistance changes in the sensors were measured using a digital source meter (Keithley 2450 digital source meter, Beaverton, OR, USA) at 50% strain after 1000 tensile release cycles.

## 3. Results and Discussion

### 3.1. Preparation and Characterization of Car-P(AA-co-AM) Hydrogel

**Figure 2:**
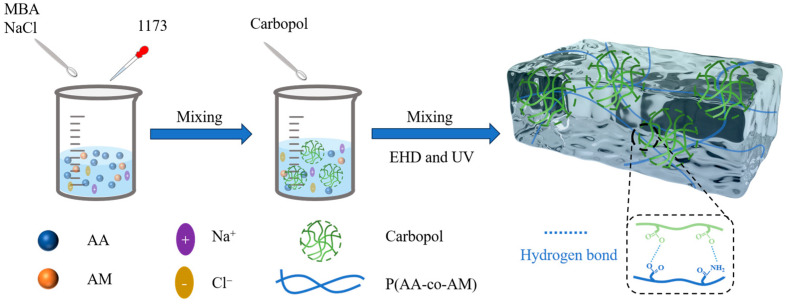
Schematic of microgel films prepared using EHD printing.

The preparation process for the Car-P(AA-co-AM) hydrogel is shown in Figure 2. A binary solvent formed by mixing water and EG was used. AA and AM were added as monomers to the solution, followed by the addition of a cross-linking agent (MBA), photoinitiator (1173), and NaCl. Carbomer 940 was added to the above solution and stirred evenly to form a stable solution such that the carbomer microgel network was spread throughout the hydrogel solution. Carbomer can not only be used as a gel matrix, but it also possesses high viscosity and shear resistance, which helps improve the rheology of the solution. Carbomer dissolved in aqueous solution formed a microgel aqueous solution with a typical microgel-like solid behavior and shear-thinning state. This kind of solid-like liquid will break the physical action and flow when subjected to sufficient stress, and will resume solid-like behavior when the stress disappears, maintaining the integrity of the printed shape. Moreover, hydrogel molecules also form a three-dimensional network that significantly increases the viscosity of the solution. These effects cause changes in the rheological behavior of hydrogel solutions. The solution was printed into a shape using EHD printing. After exposure to ultraviolet light, the AA and AM monomers were copolymerized to obtain the P(AA-co-AM) copolymer. A hydrogen-bonded cross-linked network was formed between the carboxyl group (–COOH) of AA and the amide group (–CONH_2_) of AM. Carbomer contains a large number of carboxylic acid groups (–COOH), which can form hydrogen bond crosslinks between the carboxyl and amide groups on the P(AA-co-AM) copolymer network. The two networks in the hydrogel formed dense connections, eventually forming a Car-P(AA-co-AM) hydrogel with dual physical networks. Additionally, the addition of NaCl improved the ion concentration in the hydrogel, resulting in good ionic conductivity.

**Figure 3:**
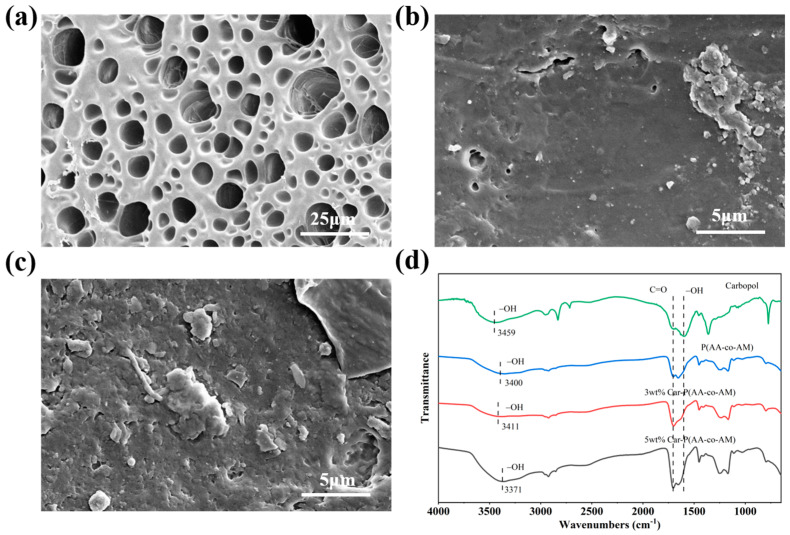
(**a**) SEM image of P(AA-co-AM) hydrogel. (**b**) SEM image of 3 wt.% Car-P(AA-co-AM) hydrogel. (**c**) SEM image of 5 wt.% Car-P(AA-co-AM) hydrogel. (**d**) FT-IR spectra of carbomer and hydrogel.

As shown in Figure 3a, the freeze-dried P(AA-co-AM) hydrogel had a three-dimensional porous network structure. Figure 3b shows an SEM image of the Car-P(AA-co-AM) hydrogel containing 3 wt.% carbomer, where the internal network morphology of the hydrogel changed significantly with the addition of the polymer carbomer. The introduction of a polymer network into the three-dimensional porous network causes its surface to adhere to numerous fiber network structures, which significantly improves the degree of cross-linking within the hydrogel. As shown in Figure 3c, when the carbomer content was increased to 5 wt.%, numerous fibers were distributed in the hydrogel network, indicating that the carbomer network and P(AA-co-AM) copolymer hydrogel network were fully cross-linked to form a denser hydrogel network [34].

FT-IR tests were performed on the hydrogel to reveal the interactions between the different components of the hydrogel, as shown in Figure 3d. The peak of the carbomer at 3459 cm^−1^ represents the characteristic peak of –OH, with the strength of this peak primarily reflecting the number of hydrogen bonds present. Both the carbomer and P(AA-co-AM) hydrogels contained numerous hydroxyl groups. When 3 wt.% of carbomer and P(AA-co-AM) hydrogel were added to form a gel, the position of the characteristic peak of –OH shifted from 3459 cm^−1^ to 3411 cm^−1^. Similarly, when the content of carbomer reached 5 wt.%, the characteristic peak of –OH shifted to 3371 cm^−1^, indicating that a significant number of hydrogen bonds were formed between the carbomer and P(AA-co-AM) copolymer. Throughout the entire process of hydrogel formation, only changes and shifts in the strength of this characteristic peak were observed, and no new characteristic peaks appeared. This indicates that only physical changes occurred during the entire process, and no chemical reactions occurred.

### 3.2. Printability of Hydrogel

**Figure 4:**
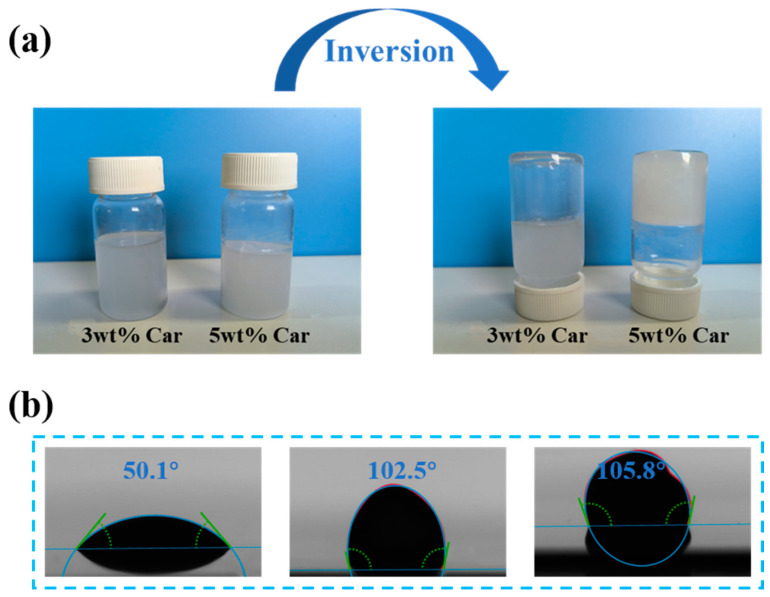
(**a**) State of hydrogel solution after being turned over. (**b**) Contact angles of hydrogel solutions containing 3 wt.%, 5 wt.%, and 7 wt.% carbomer.

Hydrogel solutions are typically dilute solutions with low viscosities; therefore, forming them directly using 3D printing is challenging [35]. Introducing a carbomer microgel can impart thixotropic properties to the solution. As shown in Figure 4a, the 5 wt.% Car-P(AA-co-AM) hydrogel solution exhibited solid-like properties and did not flow when placed upside down, whereas the 3 wt.% Car-P(AA-co-AM) hydrogel precursor solution still exhibited liquid flow properties. This quasi-solid property enables the hydrogel solution to exhibit good thixotropy and maintain its shape without collapsing during printing. Figure 4b shows the contact angle of the hydrogel solution on photographic paper substrate, which was determined using the sitting drop method. The contact angle of the 3 wt.% carbomer solution is 50.1°, indicating hydrophilic properties. When this solution is printed on photographic paper, its hydrophilic properties cause it to scatter and lose its original shape, thus making it unsuitable for 3D printing. The contact angle of the 5 wt.% carbomer hydrogel solution is 102.5°, indicating hydrophobicity. When printed on a substrate, it can maintain its original shape without collapsing, and the droplets form complete shapes, thus meeting the requirements for high-resolution printing. Although the 7 wt.% carbomer hydrogel precursor solution also exhibits a hydrophobic contact angle of 105.8°, its droplet shape is irregular owing to its high viscosity.

**Figure 5:**
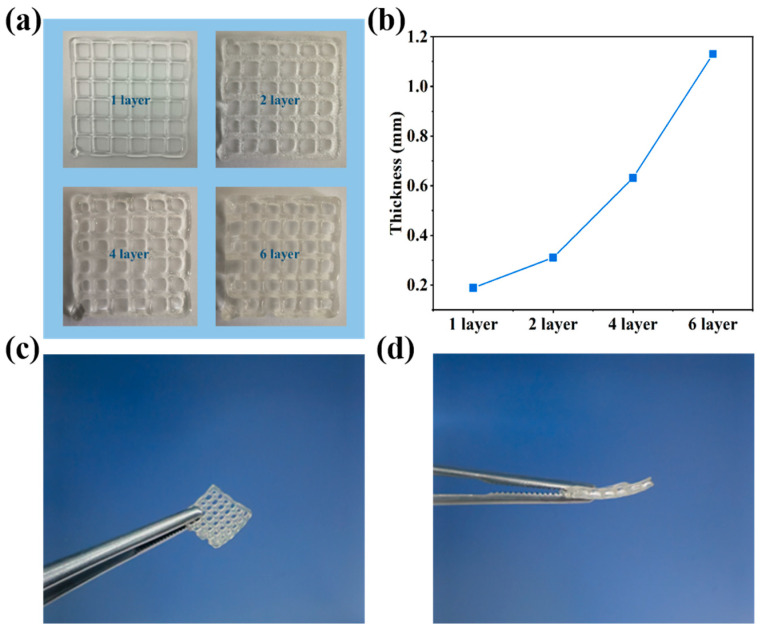
(**a**) Images of hydrogels with different numbers of layers printed using EHD printing. (**b**) Relationship between the number of printed layers and thickness of hydrogel. (**c**,**d**) Surface and cross-sectional views of hydrogels consisting of six layers printed using EHD printing.

Figure 5a shows the shape of the hydrogel printed with different layers. The thickness of the hydrogel increased with the stacking of the printed layers. As shown in Figure 5b, the thickness of the hydrogel with one printed layer is 0.181 mm, whereas that of the hydrogel with six printed layers increases to 1.112 mm. When the number of print layers is one, the hydrogel extruded from the nozzle settles on the substrate, causing a certain spreading effect. As the print layers increase, the subsequently extruded hydrogel accumulates on the previously printed gel layer. The adhesive interaction between the adjacent layers reduces the spreading behavior, thus leading to a linear increase in thickness. Therefore, the material’s thickness shows a linear growth trend with the increase in print layers, demonstrating the good 3D printability of Car-P(AA-co-AM) hydrogels. Figure 5c,d show the surface and cross-sectional images of the printed six-layer hydrogel. Its uniform surface structure and thickness make it suitable not only for flexible strain sensors, but also for flexible pressure sensors [36,37].

### 3.3. Mechanical and Electrical Properties of Hydrogels

**Figure 6:**
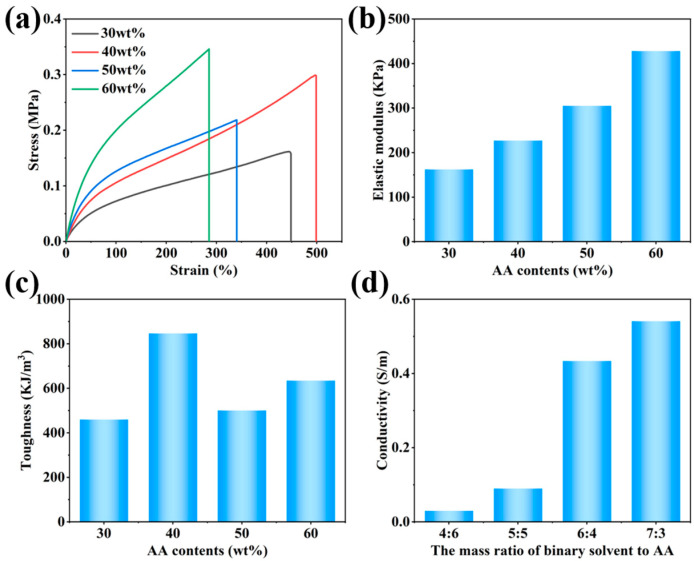
Electromechanical properties of P(AA-co-AM) copolymerization network hydrogels: (**a**) stress–strain curves, (**b**) elastic modulus, (**c**) toughness, and (**d**) ionic conductivity.

Differences in the component contents of a hydrogel often have a significant impact on its electromechanical properties. In general, increasing the number of monomers in the solution enhances the mechanical properties of hydrogels; however, an excessive number of monomers results in the formation of a dense network, which reduces the number of ion diffusion channels and leads to a decrease in conductivity. In contrast, an insufficient monomer content makes it difficult to form hydrogels with good mechanical properties. When the AA mass ratio was below 60 wt.% in the solution, hydrogels with various properties could be formed, whereas when the AA mass ratio was 70 wt.%, excessive cross-linking hindered the formation of flexible hydrogels. As shown in Figure 6a–c, the breaking elongation of the P(AA-co-AM) hydrogel decreased as the AA content increased, whereas the tensile strength increased significantly. The elastic modulus increased continuously with the increase in AA content, while the toughness did not change significantly, reaching its maximum at a content of 40 wt.%. Owing to the abundance of ions in the binary solvent, when the binary solvent constituted a high proportion of the hydrogel, the formed hydrogel exhibited higher ionic conductivity. As shown in Figure 6d, the conductivity of the hydrogel improved significantly with an increase in the mass ratio of the binary solvent. When the AA content was 40 wt.%, the hydrogel exhibited a high ionic conductivity of 0.4334 S/m. Therefore, the hydrogel containing 40 wt.% AA exhibited superior overall properties and was suitable for subsequent experiments.

**Figure 7:**
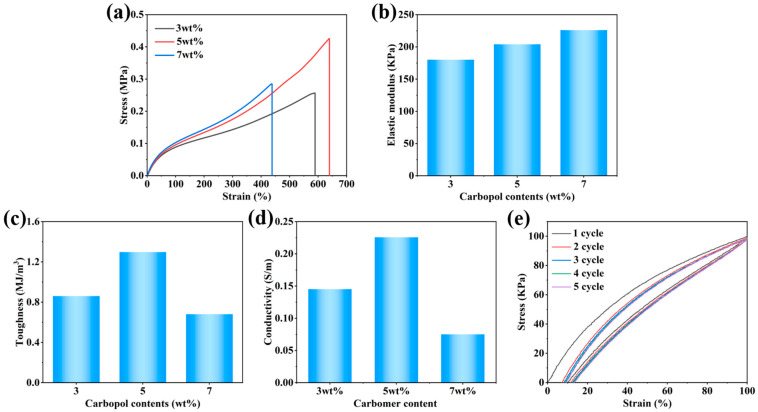
Electromechanical properties of Car-P(AA-co-AM) hydrogels: (**a**) stress–strain curves. (**b**) elastic modulus, (**c**) toughness, (**d**) ionic conductivity, and (**e**) cyclic tensile stress–strain curves.

The appropriate amount of carbomer powder was added to the hydrogel solution to adjust the rheology and satisfy the 3D printing requirements. As shown in Figure 7a, after adding 3 wt.% carbomer to the hydrogel precursor solution, the hydrogel elongation at break dramatically increased from 498.76% to 598.36%, whereas the tensile strength did not change significantly. After incorporating 5 wt.% carbomer, the elongation at break was further enhanced to 639.73%, while the tensile strength increased to 0.4243 MPa. A moderate amount of the carbomer microgel network was intensively cross-linked with the P(AA-co-AM) hydrogel network owing to hydrogen-bonding interactions, which resulted in a significant enhancement of the hydrogel’s mechanical properties. When the carbomer content reached 7 wt.%, the internal network of the gel became extensively cross-linked, resulting in a significant reduction in all gel properties. Figure 7b shows the variation in the elastic modulus of the hydrogel with more network linkages within the carbomer hydrogel, more energy dissipation modes, and a slight decrease in the elastic modulus. Figure 7c shows that the introduction of carbomer substantially increased the toughness of the hydrogel, reaching as high as 1.3 MJ/m^3^ when the carbomer content was 5 wt.%. As shown in Figure 7d, the addition of the carbomer decreased the conductivity of the hydrogel; however, it still retained an ionic conductivity of 0.2256 S/m, which meets the sensing requirements. As shown in Figure 7e, the hydrogel was subjected to five continuous loading–unloading cycles of 100% strain at a speed of 30 mm/min, demonstrating good mechanical stability.

### 3.4. Sensing Properties of Hydrogel

**Figure 8:**
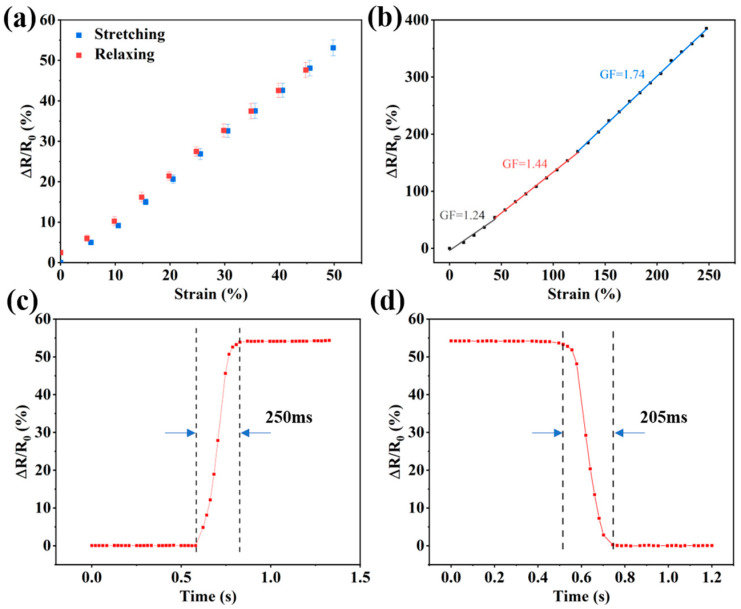
(**a**) Resistance change in sensors under 50% strain during the stretch–recovery process. (**b**) Resistance change fitting curve and sensitivity of the sensor in the 0–250% strain range. (**c**,**d**) Response and recovery times of the sensor at 50% strain.

When a strain sensor is subjected to a tensile force, its internal structure often undergoes deformation, and a lag phenomenon often occurs because of the recovery speed of the sensor not keeping up with the stretching speed, which can affect its performance. Hysteresis (*DH*) is used to represent this indicator, and it is calculated as follows:

(2) $$DH=\frac{\left|A_{L}-A_{U}\right|}{A_{L}}$$

where *A_L_* and *A_U_* are the areas under the loading and unloading curves, respectively, for Δ*R*/*R*_0_ at 50% strain. As shown in Figure 8a, the hysteresis of the hydrogel strain sensor was 4.83% at 50% strain loading, indicating that the strain sensor had low hysteresis and fast recovery when unloaded. Sensitivity is a key parameter for flexible sensors, and higher sensitivity can significantly improve the accuracy of the sensor. The sensitivity of a strain sensor is usually represented by the strain coefficient (*GF*), which is calculated using the following formula:

(3) $$GF=\frac{\Delta R/R_{0}}{\varepsilon }$$

where *R*_0_, Δ*R*, and *ε* denote the original resistance, relative resistance change, and relative strain change during stretching, respectively. As shown in Figure 8b, the flexible strain sensor exhibits a sensitivity of 1.24 in the 0–50% strain range, reaching a maximum of 1.74, and showcases the excellent linearity of resistance change, making it highly suitable for detecting strains quantitatively. The response and recovery times are important parameters for flexible strain sensors. In the field of human motion monitoring, strain sensors typically operate within the 50% strain range; therefore, their responses were tested at 50% strain. As shown in Figure 8c,d, the response and recovery times of the flexible strain sensors were 250 and 205 ms, respectively, which enabled them to respond quickly to the movements of the human body.

**Figure 9:**
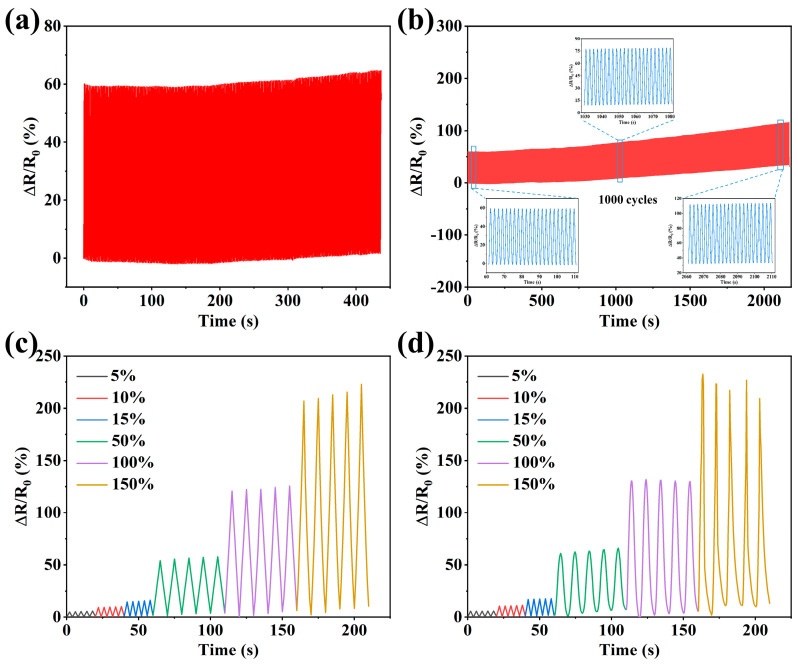
Relative resistance variation in sensors during (**a**) 200 and (**b**) 1000 tensile release cycles at 50% strain. Cyclic resistance changes in (**c**) thin-film and (**d**) mesh-type hydrogel flexible strain sensors at 5–150% strain.

The fatigue resistance of a sensor reflects its operational lifespan. The fatigue resistance of the flexible strain sensors was validated by performing multiple tensile recovery cycles. Figure 9a shows that the resistance signal of the flexible strain sensor was stable after 200 consecutive cycles under 50% strain. Owing to mechanical hysteresis, the flexible strain sensor entered the next cycle before the resistance returned to its initial value after the last stretching recovery cycle, resulting in the continuous accumulation of resistance changes and an upward shift in resistance changes. As shown in Figure 7b, when the number of stretch–recovery cycles was increased to 1000, the sensor resistance signal exhibited an upward shift; however, the percentage change in resistance remained essentially the same. The resistance change curves of the three time periods were recorded for each of the 1000 cycles, and Figure 9b shows that the resistance changes in these three time periods remained extremely stable, indicating the good stability of the flexible strain sensors. The cyclic resistance changes of the electrohydrodynamically printed thin-film-type (Figure 9c) and grid-type (Figure 9d) strain sensors were examined for small strains of 5–15% and large strains of 50–150%, respectively. Both the thin-film and grid hydrogel sensors exhibited reversible and repeatable resistance output signals. The grid-type hydrogel strain sensor exhibited a lower recovery ability than the film-type hydrogel strain sensor, owing to the loose connection between the grids; however, its sensitivity was higher. These two types of hydrogel strain sensors have their advantages and disadvantages; however, both can be used for signal transmission.

**Figure 10:**
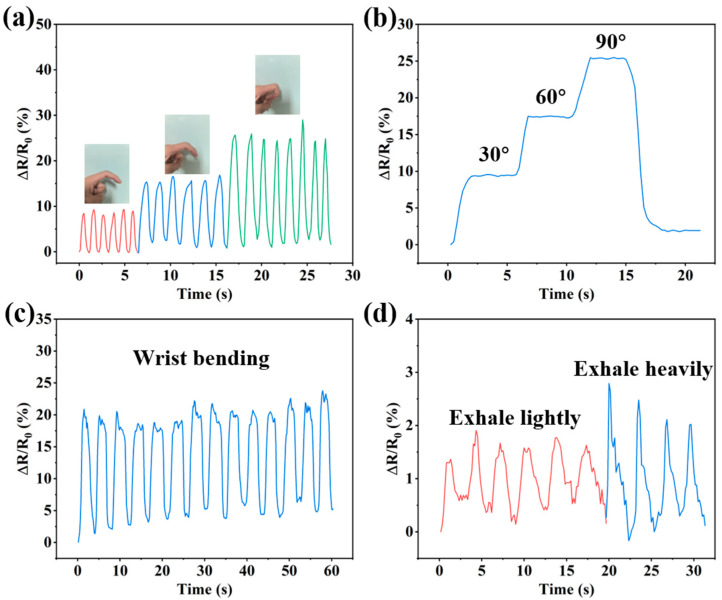
Car-P(AA-co-AM) hydrogel flexible strain sensors for human motion monitoring: (**a**,**b**) finger flexion, (**c**) wrist flexion, and (**d**) exhalation.

To monitor the movement status of the human body in real time, grid-like strain sensors were mounted on certain parts of the human body, and the change in the resistance of the sensors during the movement of these body parts was observed. Mesh hydrogel strain sensors with numerous holes provide better comfort and breathability when worn over the body. Figure 10a shows the resistance change curve of the sensor after the continuous bending of the finger at three angles, indicating that the sensor exhibited good stability and accuracy. Furthermore, as the bending angle of the finger increases, the resistance change also changes in the same proportion. Figure 10b shows the change in the resistance of the finger after remaining at each bending angle for a few seconds, indicating the sensing stability of the sensor. The sensor mounted on the wrist also had a good electrical signal response, as shown in Figure 10c, and effectively responded to and recovered from resistance changes during multiple bends of the wrist. As shown in Figure 10d, the sensor can also be used to sense small forces. When gently exhaling onto the sensor, a significant fluctuation in resistance occurred, and increasing the strength of the exhalation further increased the resistance change. This performance enables the application of the sensor in the field of respiratory sensing, thereby expanding its application range.

## 4. Conclusions

In conclusion, this study proposes the use of a green and non-polluting microgel material, carbomer, to improve the rheological properties of hydrogel precursor solutions for 3D printing. The presence of carbomer not only makes the solution 3D printable but also significantly improves the mechanical properties of the hydrogel. The hydrogel sensor demonstrates good sensitivity, linearity, stability, and a wide detection range. Moreover, it can continuously and stably output electrical signals with good stability and fatigue resistance. In practical applications, the sensor can stably monitor finger bending, wrist bending, and breathing and exhaling motions, showing promising potential for recognizing different movements of the human body. In addition, the sensor possesses good air permeability, making it ideal for use on human skin. This study provides a novel strategy for the development of printable hydrogel sensors.

## Data Availability

Data available within the article.
